# Prenatal Exposure to Perfluorinated Chemicals and Behavioral or Coordination Problems at Age 7 Years

**DOI:** 10.1289/ehp.1002026

**Published:** 2010-11-09

**Authors:** Chunyuan Fei, Jørn Olsen

**Affiliations:** Department of Epidemiology, University of California, Los Angeles, Los Angeles, California, USA

**Keywords:** behavioral problems, child, coordination disorders, maternal blood, neurodevelopment, PFOA, PFOS

## Abstract

**Objective:**

Potential neurotoxic effects of perfluorinated compounds (PFCs) have been reported in highly exposed animals, but whether these chemicals are neurotoxic in humans is not known. We therefore investigated whether prenatal exposure to perfluorooctanoic acid (PFOA) or perfluorooctane sulfate (PFOS), two of the most prevalent PFCs, are associated with behavioral or coordination problems in early childhood.

**Methods:**

We used data from the Danish National Birth Cohort, which enrolled mothers in early pregnancy, and we measured maternal blood levels of PFOA and PFOS using specimens drawn around 8 weeks of gestation. When the children reached 7 years of age, mothers completed the Strengths and Difficulties Questionnaire (SDQ, *n* = 787) and the Developmental Coordination Disorder Questionnaire (DCDQ, *n* = 526) to assess behavioral health and motor coordination of their children. SDQ scores above the 90th percentile were *a priori* defined to identify behavioral problems and DCDQ scores below the 10th percentile were defined as a potential DCD.

**Results:**

The median concentrations of PFOS and PFOA in maternal blood were 34.4 ng/mL [interquartile range (IQR), 26.6–44.5] and 5.4 ng/mL (IQR, 4.0–7.1), respectively, similar to distributions reported for populations without occupational exposure. We found no association between higher SDQ scores and maternal levels of PFOS or PFOA, nor did we see any statistically significant association with motor coordination disorders.

**Conclusion:**

The findings suggest that background levels of PFOA and PFOS are not associated with behavioral and motor coordination problems in childhood. However, effects on other developmental end points, including cognitive, attentional, and clinical mental disorders not measured in this study, cannot be ruled out.

Perfluorinated compounds (PFCs) belong to a family of man-made, fluorine-containing chemicals that have been manufactured since the 1950s. Perfluorooctanoic acid (PFOA) and perfluorooctane sulfate (PFOS) are two of the most common PFCs. They have hydrophobic and lipophobic characteristics because the fluorocarbon segment and their functional groups (sulfonate or carboxylate) can lower surface tension of water. The unique properties of PFC compounds make them useful in a wide variety of special consumer applications (stain- and water-repellent treatments) and industrial applications (surfactant and emulsifier). Humans are exposed to these chemicals through consumer articles (e.g., carpet, furniture, clothing, shampoo, cleanser, dental floss), drinking water, food, dust, and occupational settings (Guo et al. 2009; Poulsen et al. 2005; Vestergren and Cousins 2009). The widespread presence of PFCs and their long biologic half-lives for elimination of PFOA and PFOS (range, 4–5 years) in humans have raised concerns about their potential impact on human health (Olsen et al. 2007). The most vulnerable age period is expected to be early in life, especially during organogenesis and rapid neuron development.

PFOA and PFOS cross the placenta and the fetal brain barrier (Butenhoff et al. 2009; Cui et al. 2009; Fei et al. 2007). Recent experimental evidence suggests that prenatal or neonatal exposure to PFOA or PFOS correlated with behavioral anomalies in rats or mice and delayed neuromotor maturation (Butenhoff et al. 2009; Johansson et al. 2008, 2009), and these behavioral modifications appear to persist into adulthood (Johansson et al. 2008). It is likely that some of these chemicals act through cholinergic or dopaminergic mechanisms involving altered responses to nicotine or imbalanced expression of the acetylcholine/dopamine phenotype (Johansson et al. 2008; Slotkin et al. 2008). The alterations in the dopaminergic system may be a possible pathophysiological cause for attention deficit/hyperactivity disorder (ADHD) symptoms (Swanson et al. 2007). PFCs can also affect protein levels of functional importance during neuron growth and synaptogenesis (Johansson et al. 2009; Liao et al. 2008; Wang et al. 2007).

ADHD and developmental coordination disorders (DCDs)—the most common neurobehavioral diseases in young children—have become much more frequently reported during the last decades (Pastor and Reuben 2008). If this is not just related to a change in diagnosis, we need to look for other potential causes, of which new environmental exposures with increasing levels over time may be candidates. The longitudinal design of the Danish National Birth Cohort (DNBC) provides us with an opportunity to explore whether maternal levels of PFOA and PFOS early in pregnancy correlate with neurodevelopmental anomalies in the offspring. Using data from the DNBC, we found that prenatal exposure to PFCs was associated with reduced fetal growth; but using DNBC data on developmental milestones at 6 and 18 months of age, we found no significant association between *in utero* exposure to PFOA and PFOS and neurologic development (Fei et al. 2007, 2008a, 2008b).

In this study, we used two validated questionnaires [the Strengths and Difficulties Questionnaire (SDQ) and the Developmental Coordination Disorder Questionnaire (DCDQ)] to examine the association between prenatal exposure to PFOA and PFOS and behavioral, social, and motor development of children at 7 years of age.

## Methods

The detailed designs of the DNBC and the PFC study nested in the DNBC have been described previously (Fei et al. 2007; Olsen et al. 2001). Briefly, the DNBC is a nationwide cohort study with prospective data from about 100,000 pregnancies, with follow-up of the children. We took a random sample of 1,400 women from 43,045 eligible women in the cohort who provided a maternal blood sample at the first antenatal care visit, gave birth to a single live-born child without congenital malformation, and completed all four telephone interviews (two during pregnancy and two after birth). When the children reached 7 years of age, a follow-up interview was conducted in 2005–2010 using either an online questionnaire or a mailed paper questionnaire.

We assessed behavioral problems using the standardized SDQ, which comprised five domains (emotional, conduct, hyperactivity, peer, and social disorders) (Youth in Mind 2009). The SDQ is a validated tool to screen for hyperactivity and attention problems among children (Goodman 1997). Parents, mostly mothers, completed a list of 25 questions that reflected their children’s behavior in the previous 6 months. The responses for each item were coded as 0 for “not true,” 1 for “partly true,” and 2 for “very true.” For each of five subscales, scores were summed giving a range of 0–10. The scores for the four scales (emotional, conduct, hyperactivity, and peer) were further summed to generate a total difficulties score ranging from 0 to 40. The prosocial score gave a score for positive prosocial behavior, and this sum was not included in the total difficulties score. If data were missing but at least three items for a subscale were completed, a scale score was prorated (*n* = 11) based on the instructions of SDQ (Youth in Mind 2009; scoring instructions available at http://www.sdqinfo.com/ScoreSheets/e1.pdf). Children having higher scores are more likely to have behavioral problems except on the prosocial subscale, where low scores indicate a problem.

The DCDQ is a parent-reported measure developed to assist the identification of DCD in children 5–15 years of age (Wilson et al. 2007). Parents were asked to compare the motor performance of their child with that of his or her peers using a 5-point Likert scale, which provides a measure of a child’s coordination in everyday functional activities. The DCDQ consists of 15 items, and a total score range of 15–75. This questionnaire was first included in the 7-year follow-up from 2006, which explains the number of the children with missing data on this outcome.

Plasma concentrations of PFOS and PFOA were blindly measured at the 3M Toxicology Laboratory (St. Paul, MN) using high-performance liquid chromatography/tandem mass spectrometry. Quality control materials (newborn calf serum) were extracted using 30 individual solid-phase extractions to establish within-run means and SDs for two levels of control (15 ng/mL and 44 ng/mL for PFOA, 10 ng/mL and 30 ng/mL for PFOS). The coefficients of variation for the between-batch spiked control values were 3.2% and 3.5% for PFOA and 2.5% and 2.8% for PFOS, respectively. The lower limit of quantitation (LLOQ) was set at 1.0 ng/mL; values of PFOA that fell below the LLOQ were assigned a value of half the LLOQ. Detailed sampling and laboratory methods have been published (Fei et al. 2007).

We used logistic regression models in the primary analyses. We *a priori* defined the top 10% of the children with SDQ data available as having a high behavioral problem score. The cut points used were as follows: emotional symptoms ≥ 4, hyperactivity ≥ 5, conduct problems ≥ 3, peer problems ≥ 2, and total difficulties ≥ 11, which corresponded to the upper 14.3%, 14.2%, 14.6%, 15.2%, 10.4% of the respective distributions. Prosocial scores < 6 were defined to indicate social problems, corresponding to 12.1% of the children. Similar to the SDQ data, scores below the 10th percentile on developmental coordination (≤ 58) were defined as potential DCD.

PFOA and PFOS levels were categorized *a priori* into quartiles, using the lowest quartile as the reference group. Potential confounding variables included in the models were parity, maternal age, prepregnancy body mass index (BMI), smoking and alcohol consumption during pregnancy, maternal socioeconomic status (SES), sex of the child, parental behavioral problem scores during their own childhood, breast-feeding, birth year, household density, and gestational age at blood drawing. Data on breast-feeding practices were collected in the interviews at 6 and 18 months after birth and were categorized as “never breast-fed or < 3 months,” “3–5 months,” and “≥ 6 months” of breast-feeding. Although breast-feeding practices for a child in this study did not directly influence maternal PFC levels we measured, this variable may be correlated with previous breast-feeding experience and thus confound the associations between PFC exposure and behavioral problems of children.

Household density was defined as the total number of rooms divided by the total number of persons in the house. Parental behavioral problem scores were calculated from the parent’s self-report on their own childhood at the 7-year interview. (The questionnaire consisted of six items and is available at http://www.ssi.dk.) To avoid loss of the subjects, missing values on maternal weight (or height) before pregnancy were imputed (*n* = 9) according to maternal height (or weight) before pregnancy, maternal age at delivery, parity, socioeconomic status (SES), and birth weight of her baby, and prepregnancy BMI was then calculated. We assigned the median value of alcohol consumption during pregnancy (*n* = 11) or parent’s behavioral scores (*n* = 10) among all the subjects to those with missing data on these variables. Analyses were also done after excluding those with missing data on covariates, and no noteworthy changes in the results were seen. We selected potential confounders based on prior knowledge and literature or change-in-estimate principles (Maldonado and Greenland 1993). If the covariates changed the estimates < 5% when taken out of the full model, we removed them from the final model. For comparability, all scores (total difficulties score and subscales) were adjusted for the same set of covariates.

In addition to the dichotomized outcome analysis described above, we ran linear regression for the SDQ and DCD scores after data transformation. The values of the SDQ subscales and DCD score are skewed, and the residuals were not normally distributed with any data transformation. Consequently, we also categorized these scores into three to six subgroups according to the distributions and used ordinal logistic regression models for these ordinal responses. The proportional odds assumption was not violated for any of these models.

Written informed consent was obtained from all participants at recruitment. We have complied with all applicable requirements of the U.S. and/Danish regulations, and this project was approved by the University of California, Los Angeles Office for Protection of Research Subjects (06-08-023-01) and the Danish Data Protection Agency (J.Nr 2006-41-6324).

## Results

By October 2009, 787 mothers had completed the SDQ when their children reached the age of 7 years, corresponding to 65% of the eligible children whose mothers took part in the 7-year data collection (*n* = 1,282). Of these children, 537 completed the DCDQ. The mean age of the children at the response time was 7.15 years (range, 7.01–8.47). The distributions of the SDQ and DCD scores are shown in Table 1. The median concentrations of PFOS and PFOA in maternal blood were 34.4 ng/mL [interquartile range (IQR), 26.6–44.5] and 5.4 ng/mL (IQR, 4.0–7.1), respectively. Maternal PFOA levels declined with increasing parity, higher household density (fewer rooms per person), longer reported duration of breast-feeding, and birth year (Table 2). PFOA levels were lower in the older mothers (Table 2) but not after adjustment for parity (data not shown). There was a positive association between PFOA levels and prepregnancy BMI (Table 2), even after taking into account all predictors of PFC exposure presented in Table 2 (except for parent’s behavioral scores during their own childhood) (*p* = 0.05, data not shown). PFOS concentrations were strongly correlated with those of PFOA (Spearman’s correlation coefficient, *r*_s_ = 0.70 and *p* < 0.01), and PFOS showed a similar relationship with those predictors above as did PFOA, except that PFOS concentrations were statistically significantly lower in women of higher SES [see Supplemental Material, Table 1 (doi:10.1289/ehp.1002026)].

As shown in Table 3, the prevalence of behavioral problems (with a high total difficulties score) was inversely associated with parity and household density after adjustment for other covariates. Lower maternal SES and parents’ behavioral scores during their own childhood were statistically significantly associated with behavioral problems of the children at 7 years of age. We also observed that women who were obese, did not drink alcohol, smoked during pregnancy, or had breast-fed < 6 months were more likely to report a child with a high total difficulties score, although all these associations were not statistically significant. A high difficulties score was more common in boys than girls, primarily reflecting differences in the hyperactivity score. The prevalence of an elevated hyperactivity score was 18.3% in boys and 10.3% in girls.

Tables 4 and 5 show the adjusted associations between PFOA and PFOS levels and SDQ and DCD scores. The prevalence of high scores for total difficulties, emotional symptoms, conduct problems, and peer problems was greater in the fourth quartile of PFOA than in the first quartile, but adjusted odds ratios (ORs) did not indicate positive associations between these outcomes and high PFOA exposure. In contrast, women in the second or third quartiles of PFOA had statistically significantly lower odds of having a child with higher scores in total difficulties, emotional symptoms, and hyperactive, compared with women in the lowest quartile (Table 4). We did not observe any association between PFOA exposure and prosocial behavior or DCD. For PFOS, there was no clear association with behavioral problems in children (Table 5), nor did we find any significant association between exposure and prosocial behavior or DCD. Results from linear regression and ordinal regression analyses using the full scales of behavioral scores also showed no significant association with PFOA or PFOS, except for an inverse association with prosocial behavior scores (which means highly exposed women are less likely to report high scores of their children) [see Supplemental Material, Tables 2 and 3 (doi:10.1289/ehp.1002026)].

## Discussion

We found little evidence for an association between prenatal exposure to PFOA or PFOS and behavioral problems or DCDs in children at 7 years of age. The only inverse association between PFCs and prosocial scores we observed may be a chance finding. Using the same cohort, we had previously reported PFOA exposure to be related to lower birth weight, birth length, and abdominal circumference (Fei et al. 2007, 2008b).

Reported potential behavioral effects of prenatal or neonatal exposure to PFOS and PFOA at high doses in rats or mice have been abnormal spontaneous behavior, increased motor activities, and reduced habituation (Butenhoff et al. 2009; Fuentes et al. 2007a; Johansson et al. 2008). In contrast, studies in adult animals showed PFOS or PFOA exposure had no or only slight neurobehavioral effects (Fuentes et al. 2007b; Sato et al. 2009). The fetal brain is expected to be particularly vulnerable because the blood–brain barrier and detoxification capabilities are not yet fully developed. PFOS concentrations in fetal rat brains were found to be higher than in adult maternal brain (Chang et al. 2009), but much lower than serum concentrations [approximately 60–70% lower in the study by Chang et al. (2009)]. If PFC exposure has an effect on neurodevelopment, *in utero* exposure or exposure in early childhood may cause more damage to the nervous system than exposure at any other stage of development, and exposure levels during early childhood have been found to be higher than in adults (Olsen et al. 2004). Our previous reports indicated that maternal blood level provides a good indicator for the antenatal exposure as measured in cord blood (*r* = 0.74 for PFOS; *r* = 0.85 for PFOA if two outliers were not included) (Fei et al. 2007). A more comprehensive assessment of PFC exposure during childhood is of importance to fully elucidate the association between PFC exposure and child behavior.

The neurotoxic effects observed in animals were at doses several orders of magnitude higher than those in the general populations. For example, Butenhoff et al. (2009) reported that the no observed adverse effect and lowest observed adverse effect dosages of PFOS were 0.3 and 1.0 mg/kg-day for development of the nervous system in rats, which correspond to approximately 6,200 and 27,600 ng/mL for the mean maternal serum concentrations, respectively (Butenhoff et al. 2009). The findings from animal studies may not apply to humans because of extremely high doses and shorter half-lives of PFOA and PFOS in animals.

The SDQ is a brief screening device for identifying high-risk children either in epidemiologic research or clinical settings (Goodman and Goodman 2009; Koskelainen et al. 2000; Wiles et al. 2006), and its reliability and validity have been well documented (Goodman and Goodman 2009; Obel et al. 2004). The sensitivity of this score to subtle changes in neurodevelopment has also been demonstrated in studies of environmental exposures such as maternal smoking and alcohol drinking (Kelly et al. 2001, 2009; Obel et al. 2004). The parents in our study were not informed of PFC values in their blood when they provided data on their children’s behavior over the previous 6 months, and differential recall is therefore unlikely. Because of the selection criteria used for sampling the subcohort and loss to follow-up in the 7-year interview, the children included in our study appeared to be healthier, on average, than the general child population. Within the DNBC, the prevalence of behavioral problems was around 10% (Divan et al. 2008) if the scores were dichotomized as borderline/abnormal according to the recommendations of the author of the instrument (Goodman 1997); however, the prevalence was low (5–6%) when we used the same norms to classify the subset of DNBC participants included in this study. We used the top 10 percentile cutoff criterion based on our sample, which was lower than the original norms and lower than cut points used in other studies (Kelly et al. 2001, 2009). Some children with behavior within the normal range are possibly misclassified as having behavior problems. If PFCs affect only clinical diseases, such as ADHD, our study may not be large enough to detect such an association.

Using this data source, we were able to identify the expected predictors of total difficulties score, such as sex of child, parity (a proxy variable of number of siblings), prepregnancy BMI, social class, breast-feeding experience, smoking during pregnancy, and parents’ difficulties scores during childhood (Obel et al. 2009; Wiles et al. 2006). We used continuous variables or ordinal responses to gain more statistical power, but reached similar conclusions, which indicated that we have not overlooked strong associations.

The DNBC is a longitudinal birth cohort with extensive data on potential confounders. Still, a protective social environment associated with PFC exposure may mask the associations, as we can only control for the data we have. Approximately 35% of subjects did not complete the 7-year interview either because they had not reached 7 years of age or because the mothers did not respond. Mothers included in this analysis tended to be of higher SES, to have been nonsmokers during pregnancy, to have had normal prepregnancy BMIs (18.5–24.9 kg/m^2^), and to have breast-fed their child > 6 months [see Supplemental Material, Table 4 (doi:10.1289/ehp.1002026)]. PFOS concentrations of those included were systematically higher, because PFOS concentrations slightly declined over time and younger children were excluded if they had not reached 7 years of age before this analysis. We adjusted for SES to minimize selection bias. Whether loss to follow-up biased the effect measures is not known, but is not expected.

In summary, maternal plasma levels of PFOA or PFOS measured early in pregnancy were not related to behavioral problems or DCDs measured by the SDQ and DCDQ in the offspring at the age of 7 years. Studies using more sensitive indicators of neuropsychological function and including more heavily exposed populations are needed.

## Figures and Tables

**Table 1:** PFOA and PFOS levels in maternal blood and SDQ^a^ and DCDQ^b^ scores at 7 years of age in the DNBC subcohort, 1998–2002.

Exposure and outcomes	Mean ± SD	Median	IQR	Range
PFOA (ng/mL)	5.7 ± 2.4	5.4	4.0–7.1	0.5–21.9
PFOS (ng/mL)	36.1 ± 13.1	34.4	26.6–44.5	7.3–106.7
Total difficulties	5.5 ± 4.2	5	3–8	0–25
Emotional symptoms	1.6 ± 1.8	1	0–2	0–9
Hyperactivity	2.2 ± 1.9	2	1–3	0–8
Conduct problems	1.2 ± 1.2	1	0–2	0–7
Peer problems	0.6 ± 1.1	0	0–1	0–7
Prosocial behavior	8.4 ± 1.5	9	8–10	3–10
DCD	67.8 ± 8.1	70	64–74	17–75

a *n* = 787.

b *n* = 537.

**Table 2:** Characteristics of study subjects according to maternal PFOA levels (ng/mL) in quartiles.

Characteristic	< LLOQ–3.95	3.96–5.32	5.35–7.11	7.14–21.90
Maternal age at delivery (years)^a^,*	31.7 ± 4.1	31.5 ± 4.2	30.4 ± 4.2	29.7 ± 4.4
Prepregnancy BMI (kg/m^2^)^a^,^b^	23.2 ± 3.7	23.7 ± 4.0	23.8 ± 4.4	24.1 ± 4.7
Parent’s behavioral scores^a^	6.56 ± 1.56	6.48 ± 1.59	6.46 ± 1.64	6.70 ± 1.51
Gestational week at blood drawing (weeks)^a^	8.29 ± 2.05	8.16 ± 2.11	7.83 ± 2.12	7.80 ± 2.05
Parity (%)*				
0	15.3	35.5	54.8	72.1
1	53.6	41.1	30.5	17.8
≥ 2	31.1	23.4	14.7	10.2
Maternal SES (%)				
Higher	55.1	56.4	55.3	47.7
Middle	36.7	37.1	35.0	43.7
Lower	8.2	6.6	9.6	8.6
House density [rooms/person (%)]				
< 1	27.6	26.4	22.3	21.8
1	30.1	25.4	34.0	26.4
1–1.5	31.1	28.4	24.4	27.9
≥ 1.5	11.2	19.8	19.3	23.9
Alcohol consumption before pregnancy [drinks/week (%)]^b^				
0 – < 1	54.4	51.0	50.5	61.1
1–1.5	14.0	18.4	15.0	17.1
2–3	19.2	15.3	19.1	15.0
> 3	12.4	15.3	15.5	6.7
Smoking during pregnancy (%)	19.4	23.9	20.3	20.8
Sex of child [male (%)]	51.5	46.7	48.2	50.8
Birth year (%)*				
1998	4.1	6.1	9.1	15.2
1999	19.9	24.4	27.9	30.5
2000	31.1	29.4	30.5	26.9
2001	27.6	23.9	23.4	18.3
2002	17.4	16.2	9.1	9.1
Duration of breast-feeding [months (%)]*				
< 3	8.7	12.2	15.2	17.3
3–5	10.7	19.8	17.8	24.4
≥ 6	80.6	68.0	67.0	58.4

a Mean ± SD.

b Missing data: prepregnancy BMI (*n* = 19); alcohol consumption (*n* = 11).

* *p* < 0.05 (analysis of variance test for maternal age at delivery; chi-square test for parity, birth year, and duration of breast-feeding); *p* = 0.07 for house density (chi-square test).

**Table 3:** Adjusted ORs between predictors of PFCs and a high total difficulties scores (score ≥ 11) at 7 years of age.^a^

Predictor	No. of children with score ≥ 11 (%)	OR (95% CI)
Maternal age at delivery (years)		
< 25	12 (20.3)	1.00 (0.44–2.28)
25–29	36 (11.8)	1.00
30–34	23 (7.9)	0.80 (0.43–1.46)
≥ 35	11 (8.2)	1.00 (0.44–2.27)
Parity		
0	47 (13.4)	1.00
1	25 (8.9)	0.66 (0.37–1.20)
≥ 2	10 (6.4)	0.41 (0.17–0.98)*
Prepregnancy BMI (kg/m^2^)		
< 18.5	3 (12.5)	0.74 (0.19–2.95)
18.5–24.9	54 (9.8)	1.00
25.0–29.9	15 (9.9)	0.97 (0.50–1.87)
≥ 30.0	10 (15.6)	1.64 (0.73–3.67)
Alcohol consumption during pregnancy (drinks/week)		
Nondrinker	57 (13.2)	1.00
< 1	8 (6.4)	0.60 (0.26–1.33)
1 to < 2	8 (6.0)	0.52 (0.23–1.18)
≥ 2	9 (9.3)	0.99 (0.44–2.23)
Smoking during the pregnancy		
No	60 (9.7)	1.00
Yes	22 (13.2)	1.24 (0.69–2.23)
Maternal SES (%)		
Higher	35 (8.3)	1.00
Middle	34 (11.3)	1.30 (0.75–2.26)
Lower	13 (20.0)	2.62 (1.19–5.77)*
House density (rooms/person)		
< 1	28 (14.5)	1.00
1	21 (9.2)	0.61 (0.31–1.18)
1–1.5	14 (6.4)	0.40 (0.20–0.84)*
≥ 2	19 (13.0)	0.76 (0.38–1.52)
Breast-feeding (months)		
Never or < 3	16 (15.2)	1.00
3–5	22 (15.4)	1.15 (0.53–2.50)
≥ 6	44 (8.2)	0.72 (0.36–1.45)
Gestational age at blood drawing (weeks)		0.97 (0.87–1.09)
Sex		
Girls	37 (9.3)	1.00
Boys	45 (11.6)	1.32 (0.81–2.17)
Birth year		
1998	10 (14.7)	1.15 (0.48–2.78)
1999	21 (10.4)	0.83 (0.42–1.65)
2000	19 (8.2)	0.58 (0.29–1.16)
2001	22 (12.0)	1.00
2002	10 (9.8)	0.85 (0.36–2.01)
Parent’s behavioral scores during their own childhood		
5	13 (5.5)	1.00
6	14 (6.2)	1.12 (0.50–2.53)
7	21 (13.3)	2.48 (1.16–5.33)*
≥ 8	34 (20.0)	3.79 (1.87–7.70)*

CI, confidence interval.

a All variables listed in the table above and maternal PFOS concentrations (as a variable of PFC exposure) were put in the model.

* *p* < 0.05.

**Table 4:** ORs for higher SDQ and lower DCD scores at 7 years of age according to maternal PFOA levels (nanograms per milliliter) in quartiles.

	< LLOQ–3.95	3.96–5.32		5.35–7.11		7.14–21.90		
Outcome	*n*	*n*	OR (95% CI)	*n*	OR (95% CI)	*n*	OR (95% CI)	*p* for trend
Total difficulties (score ≥ 11)^a^	22	16	0.56 (0.27–1.19)	12	0.36 (0.15–0.82)*	32	0.91 (0.43–1.92)	0.88
Emotional symptoms (score ≥ 4)^a^	30	17	0.48 (0.24–0.97)*	23	0.70 (0.36–1.38)	43	1.28 (0.66–2.49)	0.21
Hyperactivity (score ≥ 5)^a^	35	23	0.51 (0.28–0.94)*	20	0.39 (0.20–0.76)*	34	0.62 (0.32–1.19)	0.15
Conduct problems (score ≥ 3)^a^	26	28	1.12 (0.60–2.07)	27	1.11 (0.58–2.13)	34	1.29 (0.67–2.52)	0.47
Peer problems (score ≥ 2)^a^	25	31	1.04 (0.56–1.94)	28	1.04 (0.54–2.01)	36	1.01 (0.52–1.99)	0.98
Prosocial behavior (score < 6)^a^	25	20	0.82 (0.42–1.61)	24	1.05 (0.53–2.06)	25	1.05 (0.51–2.18)	0.75
DCD (≤ 58)^b^	16	20	1.36 (0.63–2.95)	12	0.73 (0.30–1.81)	16	1.14 (0.46–2.81)	0.89

CI, confidence interval.

a Adjusted for parity, maternal age, prepregnancy BMI, smoking and alcohol consumption during pregnancy, SES, sex of child, breast-feeding, birth year, home density, gestational age at blood drawing, parental behavioral problem scores during their childhood.

b Adjusted for parity, maternal age, prepregnancy BMI, smoking and alcohol consumption during pregnancy, SES, sex of child, breast-feeding, birth year, home density, gestational age at blood drawing.

* *p* < 0.05.

**Table 5:** ORs for higher SDQ and lower DCD scores at 7 years of age according to maternal PFOS levels (ng/mL) in quartiles.

	7.3–26.4	26.6–34.3		34.4–44.3		44.5–106.7		
Outcome	*n*	*n*	OR (95% CI)	*n*	OR (95% CI)	*n*	OR (95% CI)	*p* for trend
Total difficulties (score ≥ 11)^a^	20	23	0.95 (0.47–1.91)	14	0.56 (0.25–1.22)	25	0.92 (0.45–1.87)	0.58
Emotional symptoms (score ≥ 4)^a^	27	25	0.87 (0.46–1.63)	23	1.02 (0.55–1.92)	31	1.01 (0.54–1.90)	0.83
Hyperactivity (score ≥ 5)^a^	28	34	1.05 (0.59–1.88)	18	0.51 (0.26–1.00)	32	0.92 (0.50–1.69)	0.39
Conduct problems (score ≥ 3)^a^	23	34	1.51 (0.82–2.77)	24	0.94 (0.48–1.80)	34	1.45 (0.77–2.72)	0.53
Peer problems (score ≥ 2)^a^	22	36	1.52 (0.82–2.82)	31	1.33 (0.70–2.53)	31	1.18 (0.62–2.27)	0.83
Prosocial behavior (score < 6)^a^	21	25	1.22 (0.63–2.33)	22	1.14 (0.58–2.27)	26	1.23 (0.63–2.39)	0.61
DCD (≤ 58)^b^	17	15	0.77 (0.35–1.71)	12	0.74 (0.32–1.75)	20	1.39 (0.65–3.00)	0.41

CI, confidence interval.

a Adjusted for parity, maternal age, prepregnancy BMI, smoking and alcohol consumption during pregnancy, SES, sex of child, breast-feeding, birth year, home density, gestational age at blood drawing, and parental behavioral problem scores during their childhood.

b Adjusted for parity, maternal age, prepregnancy BMI, smoking and alcohol consumption during pregnancy, SES, sex of child, breast-feeding, birth year, home density, gestational age at blood drawing.
